# FM-test: a fuzzy-set-theory-based approach to differential gene expression data analysis

**DOI:** 10.1186/1471-2105-7-S4-S7

**Published:** 2006-12-12

**Authors:** Lily R Liang, Shiyong Lu, Xuena Wang, Yi Lu, Vinay Mandal, Dorrelyn Patacsil, Deepak Kumar

**Affiliations:** 1 Department of Computer Science and Information Technology, University of the District of Columbia, Washington, DC, 20008, USA; 2 Department of Computer Science, Wayne State University, Detroit, MI, 48202, USA; 3 University of Hawaii, USA; 4 Department of Biological and Environmental Sciences, University of the District of Columbia, Washington, DC, 20008, USA

## Abstract

**Background:**

Microarray techniques have revolutionized genomic research by making it possible to monitor the expression of thousands of genes in parallel. As the amount of microarray data being produced is increasing at an exponential rate, there is a great demand for efficient and effective expression data analysis tools. Comparison of gene expression profiles of patients against those of normal counterpart people will enhance our understanding of a disease and identify leads for therapeutic intervention.

**Results:**

In this paper, we propose an innovative approach, *fuzzy membership test *(FM-test), based on fuzzy set theory to identify disease associated genes from microarray gene expression profiles. A new concept of FM d-value is defined to quantify the divergence of two sets of values. We further analyze the asymptotic property of FM-test, and then establish the relationship between FM d-value and p-value. We applied FM-test to a diabetes expression dataset and a lung cancer expression dataset, respectively. Within the 10 significant genes identified in diabetes dataset, six of them have been confirmed to be associated with diabetes in the literature and one has been suggested by other researchers. Within the 10 significantly overexpressed genes identified in lung cancer data, most (eight) of them have been confirmed by the literatures which are related to the lung cancer.

**Conclusion:**

Our experiments on synthetic datasets show that FM-test is effective and robust. The results in diabetes and lung cancer datasets validated the effectiveness of FM-test. FM-test is implemented as a Web-based application and is available for free at .

## Background

Microarray techniques have revolutionized genomic research by making it possible to monitor the expression of thousands of genes in parallel. As the amount of microarray data being produced is increasing at an exponential rate, there is a great demand for efficient and effective expression data analysis tools. The gene expression profile of a cell determines its phenotype and responses to the environment. These responses include its responses towards environmental factors, drugs and therapies. Gene expression patterns can be determined by measuring the quantity of the end product, protein, or the mRNA template used to synthesize the protein. Comparison of gene expression profiles in patients against their normal counterpart people will enhance our understanding of a disease and identify leads for therapeutic intervention. Several important breakthroughs and progress in the gene expression profiling of diseases have been made [1-5]. More interestingly, researchers have identified many genes that play important roles in the onset, development, and progression of various diseases. Identification of these disease genes offers a route to a better understanding of the molecular mechanisms underlying pathogenesis, a necessary prerequisite for the rational development of improved preventative and therapeutic methods.

One effective approach of identifying genes that are associated with a disease is to measure the divergence of two sets of values of gene expression. A motivating example is shown in Table 1, which records the microarray gene expression values of five genes for two groups of people that are related to diabetes [6]: five insulin-sensitive (IS) humans and five insulin-resistant (IR) humans. In order to identify the genes that are associated with diabetes, one needs to determine for each gene whether or not the two sets of expression values are significantly different from each other. The two most popular methods to measure the divergence of two sets of values are t-test [7] and Wilcoxon rank sum test [7], The statistical method t-test assesses whether the means of two groups are statistically different from each other. Given two sets *S*_*1 *_and *S*_*2*_, the t-value is calculated as



where μ_*S *_and σ_*S *_are the sample mean and standard deviation of *S*, respectively.

The limitation of t-test is that it cannot distinguish two sets with close means even though the two sets are significantly different from each other. Another limitation of t-test is that it is very sensitive to extreme values.

Another popular statistical method is Wilcoxon rank sum test, which can be used to test the null hypothesis that two sets *S*_*1 *_and *S*_*2 *_have the same distribution. We first merge the data from these two sets and rank the values from the lowest to the highest with all sequences of ties being assigned an average rank. The Wilcoxon test statistic *W *is the sum of the ranks from set *S*_*1*_. Assuming that the two sets have the same continuous distribution (and no ties occur), then *W *has a mean and standard deviation given by





where *m *= |*S*_*1*_| and *n *= |*S*_*2*_|.

We test the null hypothesis *H*_*o*_: no difference in distributions. A one-sided alternative is *H*_*a*_: *S*_*1 *_yields lower measurements. We use this alternative if we expect or see that *W *is unusually lower than its expected value μ. In this case, the p-value is given by a normal approximation. We let *N*~*N*(μ,σ) and compute the left-tail *Pr(N ≤ W) *(using continuity correction if *W *is an integer).

If we expect or see that *W *is much higher than its expected value, then we should use the alternative *H*_*a*_: first *S*_*1 *_yields higher measurements. In this case, the p-value is given by the right-tail *Pr(N ≥ W)*. If the two sums of ranks from each set are close, then we could use a two-sided alternative *H*_*a*_: there is a difference in distributions. In this case, the p-value is given by twice the smallest tail value 2*Pr(N ≤ W), if *W *< μ; or 2*Pr(N ≥ W), if *W *> μ.

Although rank sum test overcomes the limitation of t-test in sensitivity to extreme values, it is not sensitive to absolute values. This might be advantageous to some applications but not to others.

## Results

To validate our approach, first, we investigated the distribution of FM d-value on a set of synthetic datasets. Second, we conducted experiments on a synthetic dataset to study the relationship between FM-test d-value and its empirical p-value. Third, on another synthetic dataset, we studied the relationship between FM d-value and the mean difference of distributions.

### The probability distribution of FM d-value

**Figure 1:**
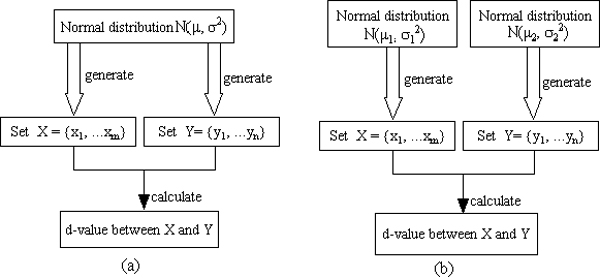
**Random generation of d-value from normal distribution**. (a) shows the random generation of two sets of values from the same normal distribution and the calculation of the FM d-value of these two sets. (b) shows the random generation of two sets of values from two different normal distributions and the calculation of FM d-value of these two sets.

**Figure 2:**
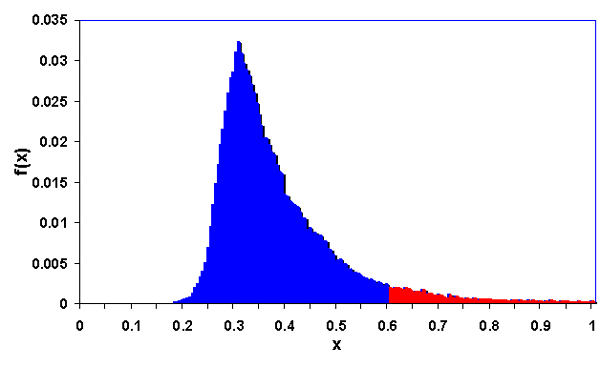
**The probability density function of FM d-value**. The probability density function of FM d-value shows that most d-values falls into the middle region and only 5% d-values are greater than 0.6058; these d-values are considered significant.

**Figure 3:**
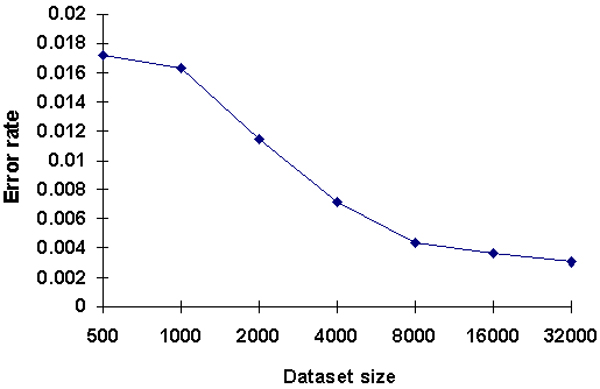
**The impact of dataset size on error rate of PDF of FM d-value**. We show the error rate for different data sizes from 500 to 32000. For each data size, we generated 10 datasets, and thus derived 10 probability density functions. The maximum standard deviation for all d-values is recorded as the *error rate *for that data size. The error rate decreases as the size of the dataset increases.

Suppose two sets *S*_*1 *_and *S*_*2 *_are randomly drawn from the same normal distribution, what is the probability distribution of FM d-value? To answer this question, we conducted the following simulation:

1. We generated *N *= 64000 pairs of sets of values, with each set containing 5 values. As shown in Figure 1(a), each value in the two data sets is randomly generated from the same normal distribution *N*(0,1).

2. We calculated the d-value for each pair of sets.

3. We then estimated the probability density value  where *δ *= 0.005. The value is essentially the fraction of the FM d-values falling in region [d-*δ*, d+*δ*] divided by the region length 2*δ*. The probability density function of the d-distribution was drawn in Figure 2.

4. At the end, in order to understand the effect of the number of pairs used for simulation, i.e., the size of the dataset, on the approximation error of the d-distribution, we generated datasets with different data sizes. For each data size, we generated 10 datasets, and thus derived 10 probability density functions. The maximum standard deviation for all d-values is recorded as the *error rate *for that data size. As shown in Figure 3, as expected, the error rate decreases as the size of the dataset increases.

From Figure 2, we can see that most FM d-values fall into the range from 0.2 to 0.5, and very few fall into the range greater than 0.6, or less than 0.2. In particular, when *d *≥ 0.6056, p-value ≤ 0.05. This is reflected in the red-shared area in Figure 2 with *f*(*x*)*dx *= 0.05. Therefore, given two sets *S*_*1 *_and *S*_*2 *_drawn from the same normal unit distribution, the chance that the pair has a FM d-value equal to or greater than 0.6056 is very low. On the other hand, if we observe that two sets have a d-value equal to or greater than 0.6056, then this is strong evidence that these two sets are drawn from two different distributions. Therefore, they should be considered as significantly divergent.

Figure 3 shows the effect of data size on the error rate of the derived probability density function. As the data size increases, the error rate decreases. We can see from Figure 3 that, after the number of pairs of sets in a dataset is greater than 8000, the trend of the error rate becomes stable. Thus, to obtain a reliable empirical p-value for FM-test, the data size should be greater than 8000.

### Relationship between FM d-value and its empirical p-value

**Figure 4:**
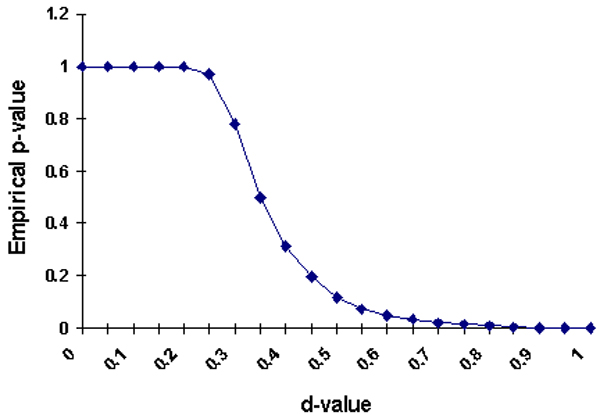
**The relationship between FM d-value and its empirical p-value**. It shows the relationship between d-value and its empirical p-value. We can see that as d-value increases, the p-value decreases. In particular, when *d *≥ *0.6056*, we have p-value ≤ 0.05.

Suppose two sets *S*_*1 *_and *S*_*2 *_are drawn from the same normal distribution, what is the probability that they have a FM d-value equal to or greater than a particular *D*? If the *D *increases, will this probability decrease? To answer these questions, we studied the relationship between FM d-value and empirical p-value as follows:

1. Based on the above experimental result, we know that we need at least 8000 pairs of sets to obtain a reliable empirical p-value. Therefore, in this experiment, we generated 10000 pairs of sets of values, with each set containing 5 values. Each value is randomly generated from the unit normal distribution *N*(0,1).

2. We calculated the d-value for each pair of sets.

3. For each pair of sets *S*_*1 *_and *S*_*2 *_with d-value *D*, we calculated its empirical p-value as *n*+1/10001 where *n *is the number of pairs in these 10000 pairs that have a d-value equal to or greater than *D*.

4. We drew the relationship between d-value and empirical p-value in Figure 4.

From Figure 4, we can see that as d-value increases, the p-value decreases. In particular, when *d *≥ 0.6056, we have p-value ≤ 0.05.

### Relationship between FM d-value and the mean difference of distributions

**Figure 5:**
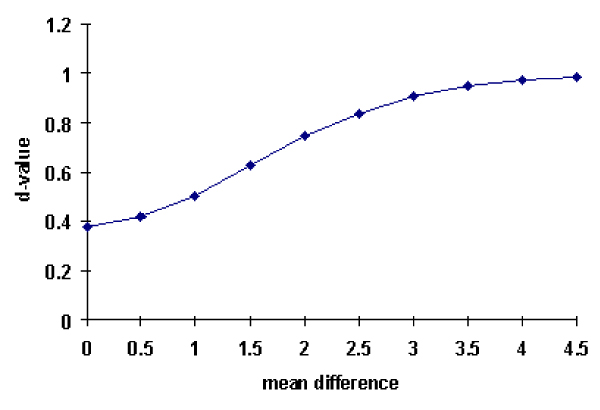
**Relationship between the mean difference of distributions and d-value**. Two datasets are generated from two distributions. Let *N*(0,1) and *N*(x, 1) be two normal distributions, where *x *is the mean difference between these two distributions. In this experiment, we consider *x *= 0, 0.5, 1, 1.5, 2, 2.5, 3, 3.5, 4, 4.5, respectively. The d-value between two sets increases when the mean difference of two data sets increases.

Suppose two sets *S*_*1 *_and *S*_*2 *_are drawn from two different distributions, then a good divergence measurement should satisfy the following property: the less overlap between these two distributions, the greater the d-value. We validated that our FM-test has this property as follows:

1. As shown in Figure 1(b), two data sets are generated from two distributions. Let *N*(0,1) and *N*(x, 1) be two normal distributions, where *x *is the mean difference between these two distributions. In this experiment, we consider *x *= 0, 0.5, 1, 1.5, 2, 2.5, 3, 3.5, 4, 4.5, respectively.

2. We generated 1000 pairs of sets of values, with the first set containing 5 values that are randomly generated from *N*(0,1), and the second set containing 5 values that are randomly generated from *N*(x, 1).

3. We calculated the d-value for each pair. Let the average of these 1000 d-values be *d*. We then plotted (*x*, *d*) in Figure 5.

4. We repeated step 2 and 3 for different *x*. Finally, the curve was drawn in Figure 5.

Figure 5 confirmed the desirable property of FM-test: the larger the mean difference between the two distributions, the greater the d-value.

## Discussion

### Analyzing diabetes data with FM-test

A diabetes dataset of microarray gene expression for a total of 10831 genes downloadable from [6] is used for analysis. For each gene, there are ten expression values, five from a group of insulin-sensitive (IS) people and five from a group of insulin-resistant (IR) people. Only the genes that have no null expression values are included in this analysis. We also require that, for a gene to be included, at least five out of its ten expression values are greater than 100. This eliminates the genes whose expression values are noisy and not reliable.

The results of FM-test are compared with the results of t-test and rank sum test. As we can seen in Table 2 although the orders of ranking are different for different methods, all three methods identify these genes as significantly differentially expressed between the IS and IR groups. Furthermore, 10 worst ranked genes in FM-test shown in Table 2 are also consistent with the result of the other two methods. However, gene *U49835 *is identified by FM-test as the 21st ranked significant gene with p-value 0.0258. Neither t-test (with p-value 0.0768) nor rank sum test (with a p-value 0.1522) identifies this gene as significant.

To study the relevance of genes in insulin metabolism and diabetes, the 10 best ranked differentially regulated genes shown in Table 2 were further searched in the published literature. Human phosphatidylinositol(4,5) bisphosphate 5-phosphatase homolog (gene *U45973*) was found to be differentially expressed in insulin resistance cases. Over-expression of inositol polyphosphate 5-phosphatase-2 SHIP2 has been shown to inhibit insulin-stimulated phosphoinositide 3-kinase (PI3K) dependent signaling events. Analysis of diabetic human subjects has revealed an association between SHIP2 gene polymorphism and type 2 diabetes mellitus. Also knockout mouse studies have shown that SHIP2 is a significant therapeutic target for the treatment of type-2 diabetes as well as obesity [8]. Csermely et al. reported that insulin mediates phosphorylation/dephosphorylation of nucleolar protein nucleolin (gene *M60858*) by stimulating casein kinase II, and this may play a role in the simultaneous enhancement in RNA efflux from isolated, intact cell nucle [9]. c-myc is an oncogene that codes for transcription factor Myc that along with other binding partners such as MAX plays an important role widely studied in various physiological processes including tumor growth in different cancers. Myc modulates the expression of hepatic genes and counteracts the obesity and insulin resistance induced by a high-fat diet in transgenic mice overexpressing c-myc in liver [10].

Max interactor protein, MXI1 (gene *L07648*) competes for MAX thus negatively regulates MYC function and may play a role in insulin resistance. In the presence of glucose or glucose and insulin, leucine is utilized more efficiently as a precursor for lipid biosynthesis by adipose tissue. It has been shown that during the differentiation of 3T3-L1 fibroblasts to adipocytes, the rate of lipid biosynthesis from leucine increases at least 30-fold and the specific activity of 3-hydroxy-3-methylglutaryl-CoA lyase (gene *L07033*), the mitochondrial enzyme catalyzing the terminal reaction in the leucine degradation pathway, increases 4-fold during differentiation [11]. Schottelndreier et al[12] have described a regulatory role of integrin alpha 6 (gene *X53586*) in Ca2+ signaling, that is known to have a significant role in insulin resistance [13].

HCGV gene product (gene *X81003*) is known to inhibit the activity of protein phosphatase-1, which is involved in diverse signalling pathways including insulin signaling [14]. Human ribosomal protein L7 (Gene *X57959*)plays a regulatory role in eukaryotic translation apparatus. It has been shown to be an autoantigen in patients with systemic autoimmune diseases, such as systemic lupus erythematosus [15]. Identification of this gene in our analysis and by [6] suggests a possible role of this gene in insulin resistance. Published reports on these genes indicate their roles in insulin signalling and warrant further investigations on their functions in insulin resistance cases. We further recommend genes *D85181, M95610 *and *U06452 *as candidate genes for future research in this area.

**Figure 6:**
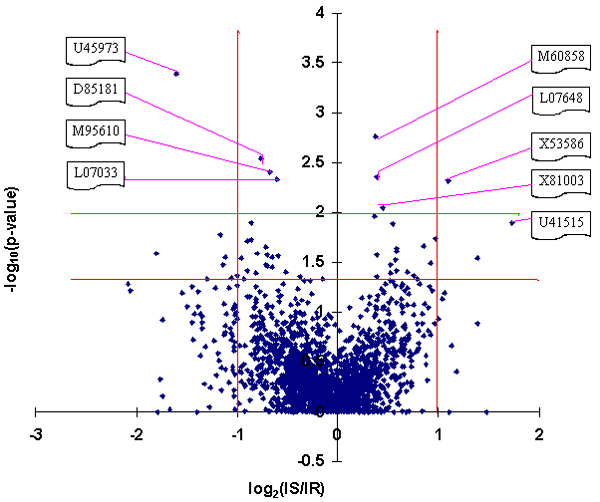
**The volcano plot for the diabetes dataset**. We compare the fold change of expression levels between the IS and IR groups to the statistical significance p-values in a volcano plot. The volcano plot arranges the genes along dimensions of biological and statistical significance. The X axis is the fold change between the two groups, which is on a log scale log_2_(/), where  is the mean of expressions in the IS group, and  is the mean of the expressions in the IR group. As we can see, a few genes shows significant difference can be visualized in the plot.

In order to compare the fold change of expression levels between the IS and IR groups to the statistical significance p-values, we presented all the genes in the diabetes dataset with a volcano plot shown in Figure 6. The volcano plot arranges the genes along dimensions of biological and statistical significance. The X axis is the fold change between the two groups, which is on a log scale log_*2*_(/), where  is the mean of expressions in the IS group, and  is the mean of the expressions in the IR group. In this way, up and down regulation appear symmetric. The Y axis represents the p-value for our FM-test, which is on a negative log scale log_10_(*p*-*value*), so that smaller p-values appear higher up. The X axis indicates biological impact of the change; the Y axis indicates the statistical evidence, or reliability of the change.

As shown in Figure 6, gene *U45973 *is identified by FM-test as the most statistically significant gene and it is over-expressed in the IR group; gene *X53586 *is identified by FM-test as the 7th statistically significant gene and it is over-expressed in the IS group. Although genes *M60858, D85181, M95610, L07648, L07033*, and *X81003 *have been identified by FM-test among the top ten significant genes, they are not over-expressed in either groups. Finally, gene *U41515 *is identified by FM-test as the 11th significant gene and it is over-expressed in the IS group.

In summary, out of the top 10 genes identified by FM-test, we could find 6 of them in the literature about their association with insulin metabolism and diabetes. Among the remaining four genes, gene *X57959 *has been recommended by [6] as a candidate gene for diabetes, we recommend that gene *D85181, M95610 *and *U06452 *could serve as candidate genes for future research in this area.

### Analyzing lung cancer data with FM-test

To study the relevance of significant genes in lung cancer, a dataset of microarray gene expression for a total of 22283 genes downloadable from [16] is used for analysis, the top ranked genes were further searched in the published literature. Most of the genes we found have a validated role in tumor progression. As showed in Table 3, we discuss a few genes that we ranked best using our method. Multiple identifiers of Keratins were ranked significant in the dataset. Cytokeratins are a polygenic family of insoluble proteins and have been proposed as potentially useful markers of differentiation in various malignancies including lung cancers [17]. Dystonin (DST/BPAG1) is a member of plakin protein family of adhesion junction plaque proteins. A recent study showed the expression of BPAG1in epithelial tumor cells [18]. Maspin (SERPINB5) was has been shown to be involved in both tumor growth and metastasis such as cell invasion, angiogenesis, and more recently apoptosis [19]. Tumor protein p73-like (TP73L/P63) is implicated in the activation of cell survival and antiapoptotic genes [20] and has been used as a marker for lung cancer. It has been suggested that the p63 genomic amplification has an early role in lung tumorigenesis [21]. CLCA2 belongs to calcium sensitive chloride conductance protein family and has been used in a multigene detection assay for Non Small Cell Lung Cancer (NSCLC) [22]. Plakophilins (PKPs) are members of the armadillo multigene family that function in cell adhesion and signal transduction, and also play a central role in tumorigenesis [23]. Desmoplakin (DSP) is a desmosome protein that anchors intermediate filaments to desmosomal plaques. Microscopic analysis with fluorescence-labeled antibodies for DSP revealed high expression of membrane DSP in Squamous Cell Carcinomas (SCC) [24]. The data analysis also identified cell cycle regulatory proteins such as CDC20 and Cyclin B1. Overexpression of CDC20 has been shown to be associated with premature anaphase promotion, resulting in mitotic abnormalities in oral SCC cell lines [25]. Mini chromosome maintenance2 (MCM2) protein is involved in the initiation of DNA replication and is marker for proliferating cells [26]. Our analysis also identified GPR87 (NM_023915) and UGT1A9 (NM_019093). Role of G protein coupled receptors are well documented in lung cancer and GPR87 could be an important gene in cancer progression. Among overexpressed genes, we suggest NM_023915 and NM_019093 as potential candidates for biological investigation.

## Conclusion

We proposed an innovative approach based on the fuzzy set theory, FM-test, that quantifies the divergence of two sets directly. We have validated FM-test on synthetic datasets and show that it is effective and robust. We also applied FM-test to a real diabetes dataset and a cancer dataset. For each dataset, we identified 10 significant genes. Within 10 significant genes in diabetes dataset, six of them have been confirmed to be associated with insulin signalling and/or diabetes in the literature, one has been recommended by others, the remaining three genes, *D85181*, *M95610 *and *U06452*, are suggested as three potential diabetes genes involved in insulin resistance for further biological investigation. Out of the 10 significantly overexpressed genes identified in the lung cancer data eight are confirmed by literature to be related to lung cancer. The remaining two genes NM_023915 and NM_019093 are potential candidates for further biological investigation. In addition, we analyzed the asymptotic properties of the distribution of FM d-value and the equation to calculate its p-value. The analysis is presented in appendix. FM-test is implemented as a Web-based application and can be accessed for free at .

## Methods

In this section, based on the fuzzy set theory [27], we present our innovative approach, the fuzzy-set-theory-based method test (FM-test), to quantify the divergence of two sets of values directly and robustly. In addition, in append ix section, we show the asymptotic property of FM-test, and then establish the relationship between FM d-value with p-value.

Let *S*_*1 *_and *S*_*2 *_be two sets of values of a particular feature for two groups of samples under two different conditions. The basic idea is to consider the two sets of values as samples from two different fuzzy sets. We examine the membership value of each element with respect to the other fuzzy set. By calculating the average of membership values, we measure the divergence of the original two sets. In particular, we perform the following steps:

1. Compute the sample mean and standard deviation of *S*_*1 *_and of *S*_*2 *_respectively.

2. Characterize *S*_*1 *_and *S*_*2 *_as two fuzzy sets *FS*_*1 *_and *FS*_*2 *_whose fuzzy membership functions, (*x*) and (*x*), are defined with the sample means and standard deviations. The fuzzy membership function (*x*)(i = 1,2) maps each value *x *to a fuzzy membership value that reflects the degree of *x *belonging to (*x*)(i = 1,2).

3. Using the two fuzzy membership functions, (*x*) and (*x*), quantify the convergence degree of two sets.

4. Define the divergence degree (FM d-value) between the two sets based on the convergence degree.

### Fuzzy Sets and Membership Functions

The sample mean μ_1 _of *S*_*1 *_is calculated as



where *n*_*1 *_is the number of elements in *S*_*1*_, and the sample standard deviation σ_*1 *_of *S*_*1 *_is calculated as



For gene 5 in Table 1, we have μ_*1 *_= 461.8, σ_*1 *_= 210.59, μ_*2 *_= 266.2, and σ_*2 *_= 45.29. We then characterize set *S*_*1 *_by a fuzzy set *FS*_*1 *_whose fuzzy membership function is defined as



The function (*x*) maps each value *x *in *S*_*1 *_to a fuzzy membership value to quantify the degree that *x *belongs to *FS*_*1*_. A value equal to the mean has a membership value of 1 and belongs to fuzzy set *FS*_*1 *_to a full degree; a value that deviates from the mean has a smaller membership value and belongs to *FS*_*1 *_to a smaller degree. The further the value deviates from the mean, the smaller the fuzzy membership value. Similarly, the fuzzy membership function for *S*_*2 *_is defined as



where μ_*2 *_and σ_*2 *_are the mean and standard deviation of *S*_*2 *_respectively.

For gene 5 in Table 1, we have  and . With these two fuzzy membership functions, the fuzzy membership values for each element with respect to the two sets can be calculated. For example, (598) = 0.81 and (598) = 2.2*E*^-12^.

### Our Proposed Method: FM-test

Since the fuzzy membership functions can overlap, one element can belong to more than one fuzzy set with a respective degree for each. For an element in *S*_*1*_, we measure the degree that it belongs to *FS*_*1 *_by applying its value to . Similarly we can apply its value to to measure the degree that it belongs to *FS*_*2*_. The idea of FM-test is to consider the membership value of an element in *S*_*1 *_with respect to *S*_*2 *_as a bond between *S*_*1 *_and *S*_*2*_, and vice versa, then the aggregation of all these bonds reflects the overall bond between these two sets. The weaker this overall bond is, the more divergent these two sets are. The strength of the overall bond between two sets is quantified by their c-value, which aggregates the mutual membership values of elements in *S*_*1 *_and *S*_*2 *_and is defined as follows.

**Definition 1 (FM c-value)**: Given two sets *S*_*1 *_and *S*_*2*_, the convergence degree between *S*_*1 *_and *S*_*2 *_in FM-test is defined as



Now we define the divergence value in FM-test (FM d-value) as follows.

**Definition 2 (FM d-value)**: Given two sets *S*_*1 *_and *S*_*2*_, the FM d-value between *S*_*1 *_and *S*_*2 *_is defined as



For gene 5 in Table 1, *c(S*_*1*_*, S*_*2*_*) = 0.326*, thus the divergence value is 1-*c(S*_1_*, S*_2_*) *= 0.674. We calculated all the p-values for the five genes in Table 1 for the three methods. One interesting observation is that, while both t-test and Wilcoxon rank sum test fail to recognize gene 5 as a significant gene since their p-values are greater than 0.05, our FM-test identifies gene 5 as a significant gene with a p-value of 0.025. The reason of the failure of t-test and Wilcoxon rank sum test is due to their sensitivity to the extreme value 141 in the first set of the gene.

Given a calculated FM d-value *D *for two sets *S*_*1 *_and *S*_*2*_, to interpret *D *in terms of "significantly divergent" or not, we need to know the cutoff value δ of *D*, so that when *D *≥ δ, the two sets are interpreted as significantly divergent. In the context of FM-test, we like to test the following null hypothesis *H*_*o*_: *S*_1 _and *S*_2 _originate from the same distribution. Then the p-value is defined as the probability {*Pr(d(S*_*1*_, *S*_2_*) *≥ *D *| *S*_*1 *_and *S*_*2 *_were randomly sampled from the same distribution}. As a convention of statistical analysis, if *p-value *≤ 0.05, then this is strong evidence to reject the null hypothesis, and accepts that the two sets are significantly divergent, while the p-value reflects the significance. It has been very common to use Monte Carlo procedures to calculate the empirical p-value which approximates the exact p-value without relying on asymptotic distributional theory or on exhaustive enumeration. Davison and Hinkley [28] present the formula for obtaining an empirical p-value as *(n+1)/(N+1)*, where *N *is the number of samples in the data set, and *n *is the number of those samples which produce the statistical value greater than or equal to the specified value.

We perform the following steps to calculate the p-value of two sets *S*_*1 *_and *S*_*2 *_with their FM d-value *D*: (1) Estimate the distribution that *S*_*1 *_and *S*_*2 *_are drawn from a normal distribution *N*(μ,σ), where μ and σ are estimated using the sample mean and standard deviation of *S*_*1 *_∪ *S*_*2*_; (2) Randomly draw *N *pairs of sets from *N*(μ,σ), then calculate the FM d-value for each pair; (3) Calculate the empirical p-value as *(n+1)/(N+1)*, where *n *is the number of pairs whose FM d-values are equal or greater than *D*.

## Authors' contributions

LRL and SL designed the algorithm and coordinated the project. XW proved the asymptotic property of FM-test and wrote part of manuscript. YL carried out the study and drafted the manuscript. VM implemented the Web-based application of FM-test. DP and DK analyzed gene functional data and wrote part of manuscript.

## APPENDIX

### Asymptotic Characteristics of the FM d-value

The FM d-value is defined in Method section as follows:



Here we are trying to establish the asymptotic characteristics of the FM d-value by estimating its corresponding mean and variance. To the end, formula (10) is rewritten by defining an indicator variable (·) as follows:



where *S *= *S*_1_∪ *S*_2 _= {*x*_*i*_,*i *= 1,..., *n*_1 _+ *n*_2_}, *n*_1 _= |*S*_1_| · *n*_2 _= |*S*_2_| and (*x*) = 1 if *x *∈ *S*_*i *_and 0 otherwise for *i *= 1,2.

Let  w.r.t. a r.v. *X *over sample space *S *with a probability *p *of choosing a sample *x *from *S*_1_. The calculation of the d-value for a given sample *x *is therefore given by *d*(*S*_1_,*S*_2_) = = 1 -. Next, the mean and the variance of Δ(*X*) are calculated respectively preparing for establishing the asymptotic distribution of the d-value.

#### (1). Calculation of the mean of Δ(*X*)

The mean of Δ(*X*) is given by





Similarly, 

By (12)–(14), the mean of Δ(*X*) when *p *= 0.5 is



#### (2). Calculation of the variance of Δ(*X*)

Since *S*_1 _and *S*_2 _are independent, the variance of Δ(*X*) is given by



Similarly,



Therefore, when *p *= 0.5



As illustrated in the beginning, d-value is a function of  which is given by *d*(*S*_1_,*S*_2_) = 1 -. By calculating the mean and the variance of Δ(*X*) in formula (Δ1) and (Δ2), the mean and the variance of the d-value are derived straightforward as follows:





For a large sample, by the **central limit theorem**, the distribution of the d-value follows a truncated normal distribution approximately: *d*(*S*_1_,*S*_2_)→ *N*(*E*(*d*),*Var*(*d*)) on a restrained domain of [0 1].

For the purpose of further illustration, several special cases of the distribution of d-value under application-specific constrains are demonstrated.

i. Balance study: *p *= 0.5, *n*_1 _= *n*_2 _= *n*/2





ii. Balance study with equal mean: *p *= 0.5, *n*_1 _= *n*_2 _= *n*/2, μ_1 _= μ_2_





iii. Balance study with equal variance: *p *= 0.5, *n*_1 _= *n*_2_, σ_1_^2 ^= σ_2 _^2 ^= σ^2^



iv. Balance study with equal variance of 1 and large samples:σ^2^= 1, *n*_1 _= *n*_2 _≥ 25



v. Balance study with equal variance of 1 and equal mean for large samples:σ^2 ^= 1, μ_1 _= μ_2_, *n*_1 _= *n*_2 _≥ 25

*E*(*d*(*s*_1_,*s*_2_)) = 1- ≈ 0.293, *var*(*d*(*s*_1_,*s*_2_))=(,)/*n *≈ 0.327/*n*

*d*(*S*_1_,*S*_2_) → *N*(0.293,0.327/*n*) with a restrained domain of [0 1].

**Figure 7:**
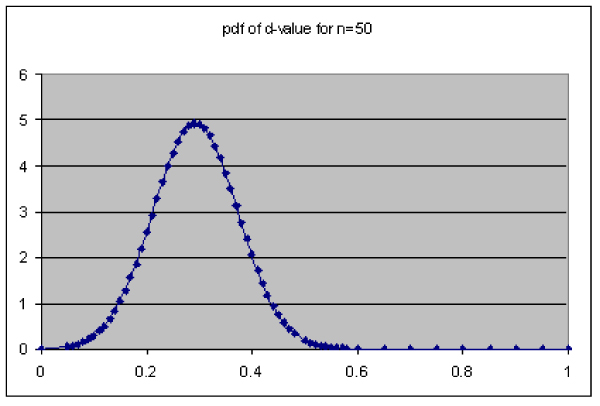
Asymptotic density function of d-value for a balance study with equal variance of one.

Figure 7 shows the density function of d-value for this special case when n = 50 with mean 0.293 and variance 0.08.

### Calculation of p-value

P-value is also called the observed level of significance and is commonly used to report the smallest α-level at which the observed test result is significant. In this section, we derived the parametric calculation of p-value for the FM test based on the asymptotic distribution obtained from section *I*.

The null hypothesis of the test is *H*_0_:μ_1 _= μ_2_, where μ_1 _and μ_2 _are the mean gene expression levels of two studied groups. According to the asymptotic distribution of the d-value, following its special case (ii) (balance study with equal mean), a test statistic under the null hypothesis for large sample size (n >= 25) is given by



Where  and .

Suppose *d*_*obs *_is an observed d-value for a given study based on two independent samples *S*_1 _= {*x*_*i*_,*i *= 1,...,*n*_1_} and *S*_2 _= {*y*_*i*_,*i *= 1,...,*n*_2_}. The population variances σ_1_^2 ^and σ_2_^2 ^are estimated by the corresponding sample variances  and . Thus the mean and variance of d-value are estimated by

 and 

P-value is therefore derived as follows:



### Application in Gene Expression Analysis

Table 4 shows the calculated P-values for the study example. It is concluded that the p-values calculated by (Δ3) are consistent with the empirical p-values listed in Table 1 except the Gene 5 which is above 0.05. As a reminder, while the formula (Δ3) is being applied for the calculation of p-values, a large sample size (n >= 25) is desired for a robust estimation due to the assumption of the CLT.

## Figures and Tables

**Table 1:** The gene expression values for five genes under two conditions.

Gene ID	IR					IS					d-value	p-value		
												
												FM	t-test	rank sum
1	750	559	649	685	636	310	359	135	97	178	0.999	0.001	0.008	0.000
2	123	142	11	406	220	305	398	707	905	688	0.756	0.012	0.011	0.031
3	246	213	232	134	67	86	79	77	94	61	0.725	0.017	0.021	0.098
4	200	191	220	83	197	49	81	116	111	135	0.708	0.019	0.024	0.058
5	598	424	695	451	141	342	260	266	229	234	0.674	0.025	0.077	0.152

Five sample genes contain two set of gene expression for two groups of people: five insulin-sensitive humans (IS) and five insulin-resistant (IR) humans. Each set of gene expression contains five gene expression values. Four values are calculated for each gene: d-value, p-value for FM-test, p-value for t-test, and p-value for rank sum test.

**Table 2:** Ten best-ranked and worst-ranked genes of diabetes identified by FM-test.

**Probe Set**	**Gene Description**	**d-value**	**Empirical p-value**	**t-test p-value**	**rank sum p-value**
U45973	Human phosphatidylinositol (4,5) bisphosphate	0.999	0.0003	0.0001	0.0076
M60858	Human nucleolin gene	0.935	0.0016	0.0017	0.0076
**D85181**	Homo sapiens mRNA for fungal sterol-C5-desaturase homolog	0.892	0.0028	0.0029	0.0147
**M95610**	Human alpha 2 type IX collagen (COL9A2) mRNA	0.872	0.0038	0.0066	0.0076
L07648	Human MXI1 mRNA	0.858	0.0043	0.0052	0.0076
L07033	Human hydroxymethylglutaryl-CoA lyase mRNA	0.855	0.0046	0.0054	0.0076
X53586	Human mRNA for integrin alpha 6	0.851	0.0047	0.0075	0.0076
X81003	Homo sapiens HCG V mRNA	0.791	0.0089	0.0077	0.0076
**X57959**	ribosomal protein L7	0.767	0.0108	0.0109	0.0313
**U06452**	melan-A	0.756	0.0126	0.0118	0.0311

X82324	POU domain, class 3, transcription factor 4	0.206	0.9987	0.407	1
M14764	nerve growth factor receptor (TNFR superfamily, member 16)	0.204	0.9989	0.652	1
M64673	heat shock transcription factor 1	0.204	0.9990	0.652	0.844
U20657	ubiquitin specific peptidase 4 (proto-oncogene)	0.197	0.9993	0.642	0.844
D17793	aldo-keto reductase family 1, member C3	0.196	0.9999	0.471	0.839
D78014	dihydropyrimidinase-like 3	0.194	1	0.620	0.548
AB002314	PDZ domain containing 10	0.191	1	0.367	0.545
L20348	oncomodulin	0.181	1	0.405	0.544
D50063	proteasome (prosome, macropain) 26S subunit	0.179	1	0.544	0.421
Z79581	H.sapiens LAZ3/BCL6 gene, first non coding exon	0.179	1	0.545	0.407

**Table 3:** Ten best-ranked (overexpressed) cancer genes identified by FM-test.

**Probe Set**	**Gene Description**	**p-value**
NM_173086	KRT6E: Keratin 6E	0.000125
NM_001723	DST: Dystonin	0.000125
NM_002639	SERPINB5: Serpin peptidase inhibitor, clade B (ovalbumin), member 5	0.000125
AB010153	TP73L: Tumor protein p73-like	0.000125
NM_023915	GPR87: G protein-coupled receptor 87	0.000125
NM_006536	CLCA2: Chloride channel, calcium activated, family member 2	0.000125
NM_001005337	PKP1: Plakophilin 1 (ectodermal dysplasia/skin fragility syndrome)	0.000125
AF043977	CLCA2: Chloride channel, calcium activated, family member 2	0.000125
NM_004415	DSP: Desmoplakin	0.000125
NM_019093	UGT1A9: UDP glucuronosyltransferase 1 family, polypeptide A9	0.000125

**Table 4:** P-values given by FM-test for five genes from the study example.

Gene ID	IR					IS					d-value	p-value			
												
												FM-test by(Δ 3)	FM	t-test	rank sum
1	750	559	649	685	636	310	359	135	97	178	0.999	0.000	0.001	0.008	0.000
2	123	142	11	406	220	305	398	707	905	688	0.756	0.007	0.012	0.011	0.031
3	246	213	232	134	67	86	79	77	94	61	0.725	0.041	0.017	0.021	0.098
4	200	191	220	83	197	49	81	116	111	135	0.708	0.014	0.019	0.024	0.058
5	598	424	695	451	141	342	260	266	229	234	0.674	0.062	0.025	0.077	0.152
